# A Double-Edged Sword Role of Cytokines in Prostate Cancer Immunotherapy

**DOI:** 10.3389/fonc.2021.688489

**Published:** 2021-11-16

**Authors:** Chenyu Mao, Yongfeng Ding, Nong Xu

**Affiliations:** Department of Medical Oncology Center, The First Affiliated Hospital, College of Medicine, Zhejiang University, Hangzhou, China

**Keywords:** prostate cancer, cytokine, immunotherapy, tumor microenvironment, anti-tumor

## Abstract

Prostate cancer (PC) is one of the most common malignancies among men and is the second leading cause of cancer death. PC immunotherapy has taken relatively successful steps in recent years, and these treatments are still being developed and tested. Evidence suggests that immunotherapy using cytokines as essential mediators in the immune system may help treat cancer. It has been shown that cytokines play an important role in anti-tumor defense. On the other hand, other cytokines can also favor the tumor and suppress anti-tumor responses. Moreover, the dose of cytokine in cancer cytokine-based immunotherapy, as well as the side effects of high doses, can also affect the outcomes of treatment. Cytokines can also be determinative in the outcome of other immunotherapy methods used in PC. In this review, the role of cytokines in the pathogenesis of cancer and their impacts on the main types of immunotherapies in the treatment of PC are discussed.

## Introduction

Prostate cancer (PC) is one of the most common malignancies among men, affecting approximately 1,600,000 people worldwide each year, killing more than 300,000 people (1). According to the latest statistics, the number of new cases of prostate cancer in 2020 is about 1,414,259, and the number of deaths due to this cancer is 375,304 (2). Prostate malignancy occurs in several stages, including intraepithelial neoplasia (PIN), localized PC, advanced prostate adenocarcinoma with local invasion, and finally, metastatic PC form (3, 4). Evidence suggests that sex hormones play an important role in the pathogenesis and progression of PC so that in castrated men, tumors progressed less (5). For this reason, androgen deprivation therapy (ADT)-based therapy has been able to prevent the progression of this type of cancer, although there is resistance against it in some cases, and the treatment fails (3). Studies show that some tumor cell subsets in PC can modify the expression or function of their androgen receptors, leading to the growth and survival of these cells even in the absence of androgens. Regrettably, this tumor behavior alteration usually can cause disease relapse upon androgen ablation (6–8).

On the other hand, chronic and dysregulated inflammation following infections or autoimmune disorders can also cause prostate cell malignancy, although the exact mechanism is unknown (9, 10). Studies showed that prostatic acid phosphatase (PAP), prostate-specific antigen (PSA), prostate-specific membrane antigen (PSMA), prostate stem cell antigen (PSCA), T-cell receptor gamma alternate reading frame protein (TARP), transient receptor potential (trp)-p8, and six-transmembrane epithelial antigen of the prostate (STEAP) are the main prostate-specific proteins and the cellular and humoral immune system can respond to these antigens (11–14).

The immune system and its components, such as cytokines and chemokines, play an essential role in the host defense processes (15). In cancer, different immune cells and associated mediators can have pro- or anti-tumor properties (16). Cytokines are small proteins between 10 to 80 kDa and act as mediators due to cell communications and various other biological processes (17). Several immune and non-immune cells such as lymphocytes, macrophages, fibroblasts and even tumor cells can produce and release cytokines (18). Because cytokines play an essential role in the immune system’s response, failure to produce or over-produce them can lead to various disorders and diseases (19). In cancer, cytokines can physiologically play anti-tumor and pro-tumor properties (20).

Consequently, cytokines have also been shown to be used in cancer immunotherapy, although this method has always had several challenges, including effective dose adjustment and ineffective monotherapy using cytokines (21). On the other hand, anti-inflammatory mediators and immune cells with inhibitory phenotypes in the tumor microenvironment (TME) have always been a significant challenge for cancer researchers. Following some immunotherapy-based methods, such as chimeric antigen receptor (CAR) T-cell therapy or monoclonal antibody therapy, side effects such as cytokine release syndrome (CRS) occur due to the dysregulated production and release of inflammatory cytokines (22). As a result, cytokines can play a dual role in cancer immune responses and cancer immunotherapy outcomes. This review focuses on the latest findings on the beneficial and destructive effects of cytokines following PC immunotherapy, as well as the challenges of using them in the treatment of PC.

## Immunobiology of PC

Immune system responses directly related to resident and infiltrated cells in the PC TME and secreted mediators can determine tumor clearance or tumor growth and treatment outcome. Among infiltrated cells, M1 macrophages, neutrophils, dendritic cells (DCs), and tumor-infiltrating lymphocytes (TILs) can participate in the anti-tumor defense. However, the inhibitory phenotype of these cells, such as regulatory T cells (Tregs), along with myeloid-derived suppressor cells (MDSCs) and anti-inflammatory mediators, leading to tumor progression through suppression of anti-tumor immune responses (23). Evidence suggests that in several cancers, the accumulation of T helper cells and cytotoxic T lymphocytes (CTLs) cells at the periphery of tumor tissue can be associated with a satisfactory prognosis (24). Studies have shown that the presence of CTLs (CD8^+^ TILs) in the tissue of solid tumors is associated with a better prognosis due to their cytotoxic and anti-tumor activity, while in PC, this has not yet been fully established, and some studies have reported that increase of CTLs in the prostate TME has led to worsening of the condition in patients (25–27). The reason is the positive association of CD73 expression as an immunosuppressive molecule with the increase in CTLs (27). Examining the characteristics of infiltrated CTLs in the TME of patients with PC showed that their cytotoxic and anti-tumor activity is significantly reduced. However, it is not yet fully understood why CTLs are exhaustive, anergic and senescent in this pathologic state (28). As noted, the balance between inflammatory and anti-inflammatory responses in the TME can determine the fate of the tumor, treatment, and patient survival. The cytotoxic activity of infiltrated CTLs in tumor tissue in PC can be affected by anti-inflammatory and immunosuppressive mediators and critically reduce the effectiveness of anti-tumor responses, which can lead to tumor progression (29). Investigation of the TME in PC shows that immune cells with inhibitory phenotypes such as CD4^+^ forkhead box P3 (Foxp3^+^) and CD8^+^ FOXP3^+^ Tregs accumulate within the epithelial compartment the tumor margin (30, 31). Also, biopsy studies of people with PC have shown that the infiltrated TILs in the tumor tissue are often FOXP3^+^ which are associated with a bad prognosis (31). These findings show that the TME in PC is different from other tumors such as gastric cancer because, in gastric cancer, an increase in infiltrated CD8^+^Foxp3^+^ TILs is associated with a favorable prognosis (32). However, it is not only lymphocytes that infiltrate the TME, but other immune cells, such as tumor-associated macrophage (TAM), CD19^+^IL-10^+^ B cells (regulatory B cells), and MDSCs, can also have immunosuppressive activity in PC, resulting in tumor invasion and progression (32, 33). *In vitro* studies have shown that co-culture of naïve monocytes with PC tumor cells reduces the expression of co-stimulatory molecules and the activity of endocytosis of monocytes. Simultaneously, colony-stimulating factor (CSF)-stimulated monocytes have inflammatory and anti-tumor properties (32). Moreover, the production and secretion of anti-inflammatory cytokines such as tumor growth factor-β (TGF-β) and IL-10 by immunosuppressive immune cells can help inhibit anti-tumor responses in PC and have a poor prognosis in these patients (32, 34).

## Involved Cytokines in the Pathogenesis of PC

Studies on PC show that in the early stages of metastasis, cell-cell adhesion and extracellular matrix cells (ECMs) are reduced following changes in the expression of specific genes such as *αVβ3* or *α2β1* in tumor cells and facilitate the metastasis process (35). On the other hand, mediators secreted by prostate tumor cells, stromal cells, and immune cells in the TME alter adhesion proteins’ expression and led to chemotactic-mediated immune cell infiltration, destroying the basement membrane and ECM. Most of these mediators are inflammatory and anti-inflammatory cytokines that can facilitate various processes leading to tumor removal or progression in an autocrine and/or paracrine manner (36). Moreover, in metastatic PC, numerous circulating tumor cells (CTCs) can express mesenchymal (vimentin, N-cadherin and O-cadherin), stem cells (CD133), and epithelial cell (E-cadherin, epithelial cell adhesion molecule (EpCAM), and cytokeratins) markers (37). Cytokines such as IL-6, IL-7, IL-8 and TGF-β can facilitate the epithelial-mesenchymal transition (EMT) process, which is an essential phenomenon in cancer induction metastasis (38–42). Among these cytokines, IL-6 can stimulate the expression of matrix metalloproteinases (MMPs) by fibroblasts in the TME *via* inducing EMT, angiogenesis, and tumor growth (43). Neuroendocrine differentiation is realized in some subtypes of PC that may be partially stimulated by androgen ablation. A similar effect has been reported upon cytokine therapy of cells with IL-10. Furthermore, IL-23 secreted by myeloid cells can also play a role in regulating the androgen response. Studies showing a link between cytokines, especially IL-6, and androgens have shown that IL-6, which acts through its transcription factor (STAT) pathway, may be involved in enzalutamide (a nonsteroidal antiandrogen) resistance. Activation of the STAT-3 pathway is accompanying augmented cell stemness. Moreover, activation of the androgen receptor by IL-6 in some subtypes of PC is associated with increased growth of tumor cells *in vitro* and *in vivo*. It has been reported that some molecules such as niclosamide and gallilactone can be very effective in simultaneously inhibiting the STAT3 pathway as well as the androgen receptor (44). The synergistic effect of TGF-β and epidermal growth factor (EGF) has also been shown to lead to the metastasis of tumor cells to the bone in PC. TGF-β can also inhibit anti-tumor responses, reduce the expression of class I major histocompatibility complex (MHC) by tumor cells and escape the immune system, as well as induce the EMT process (45, 46).

All of these occurrences ultimately lead to tumor growth, invasion, and metastasis in PC. One of the most important and essential events in tumor cell metastasis is angiogenesis, which causes cancer invasion and progression through the supply of oxygen and nutrients, and PC is no exception to this rule (47). In this critical phenomenon, cytokines including vascular endothelial growth factor (VEGF), TGF-β, and IL-8 also play an active role by induction of angiogenesis through increasing the proliferation and migration of endothelial cells (48). However, among the mentioned cytokines, VEGF has a more significant role with extra mitogenic properties for endothelial cell proliferation (49). Therefore, it seems that proangiogenic cytokines, particularly VEGF, are involved in the invasion and metastasis of PC. TGF-β is also involved in angiogenesis because it can induce angiogenesis and tumor cell metastasis in PC by regulating IL-8 expression (50). To confirm this, studies showed that by blocking and inhibiting the TGF-β/TGF-β receptor II, angiogenesis and prostate tumor cell metastasis were considerably reduced. In contrast, treatment of tumor cells with TGF-β increased the process of angiogenesis and tumor cell metastasis in animal models (50, 51).

Additionally, IL-6, TGF-β, VEGF, receptor activator of nuclear factor-kappa-β ligand (RANKL), and some chemokines participate in forming premetastatic niche, CTC attachment to endothelial, stimulation of extravasation, TME remodeling, and establishment of viable macro-metastasis (52, 53). Osteoclastogenesis and bone resorption are the main bone microenvironment remodeling processes in metastatic PC that occur following the establishment of tumor cells in the bone tissue as secondary tissue (54, 55). Previous studies revealed that IL-6, TGF-β stromal cell-derived factor 1 (SDF-1), RANKL, and CCL2 produced by bone stromal and tumor cells are responsible for this alteration in the attacked bone tissue (56–59).

Interestingly, contrary to our lessons from the past, the theory suggested that inflammation may play a role in PC formation (60). In this context, a study on human PC (LNCaP) cell line showed that tumor necrosis factor-α (TNF-α) and IL-17 could induce protein expression of programmed cell death protein ligand 1 (PDL-1) on the surface of tumor cells as an inhibitory molecule that is involved in CTLs suppression through activation of NF-κB signaling pathway (61). Investigations demonstrated that in PC and benign prostatic hyperplasia (BPH), the expression of IL-1α and IL-1RI in epithelial cells was up-regulated and associated with cell proliferation and high prostate-specific antigen levels (60). Furthermore, IL-17 can stimulate the production of induced cyclooxygenase-2 (COX-2) expression in the epithelial cells of BPH tissue (62). Some studies suggested that IL-1α and IL-6 might be two critical inductors to assess malignancy because these types of inflammatory cytokines were associated with a bad prognosis in patients with PC (63, 64). In this context, previous investigations showed that plasma levels of IL-6 were significantly raised in patients with metastatic PC (65, 66). Also, the findings showed that the levels of IL-6 soluble receptor (IL-6sR) were higher in the plasma of PC patients with bone metastasis than in non-metastatic PC patients and healthy subjects (64). The findings of another investigation demonstrated that IL-1α induces the immunosuppressive function of mesenchymal stem cells (MSCs), escaping PC cells from anti-tumor immune responses (67). It appears that due to the multiple effects of inflammatory cytokines, both inflammatory and anti-inflammatory in different contexts and the TME signals can play both protumor and antitumor roles (68–70). *In vitro* studies on PC cell lines have shown that tumor cells with IL-6 increase androgen receptor (AR) expression and function on these cells, affecting many convenient cellular processes (71). In the case of TNF-α, the results are also very contradictory because, in different cancers, it can have both apoptotic and survival impacts on tumor cells, and more studies are needed to find out the exact role of this cytokine. It can also act in the same way in PC with other inflammatory cytokines such as IL-6 (60). The evidence showed a cross-talk between the immune system and sex hormones such as androgens, estrogen, and progesterone (72). Among these hormones, estrogen could be involved in inflammatory mechanisms *via* stimulation of interferon g (IFN-γ) production and activation of lymphocytes as the main anti-tumor immune cells in PC (73). Study on the differential TNF-α and IFN-γ expression between prostate hyperplasia and PC showed that the TNF-α and IFN-γ had a higher expression in PC cells than prostate hyperplasia. However, the function of these cytokines should be investigated in the pathogenesis of prostate lesions (74). It has also been shown that estrogen can affect both subsets of T cells, Th1 and Th2. It can induce the trafficking of CD4^+^ Th1 cells and overexpression of IL-4, IL-13, and TGF-β by Th2 cells in BHP nodules. IL-4 and IL-13 are able to stimulate prostate epithelial cells to express3β-hydroxysteroid dehydrogenase/Δ⁵⁻⁴ isomerase (3b-HSD), which is involved in the generation of the active form of androgens (75). It has been shown that following the secretion of inflammatory cytokines from immune cells, BPH epithelial cells, stromal cells, and hypoxia in the TME, the demand for oxygen consumption by infiltrated cells increases. These factors eventually cause these factors tissue damage (76, 77). In PC, cytokines and growth factors secreted in the TME affect infiltrated immune cells and stromal and epithelial cells (77).

Moreover, infiltrated chronically activated T cells, B cells, and macrophages in the BPH tissue are responsible for producing and secreting TGF-β, IL-2, and IFN-γ (78). Additionally, it has been revealed that in BPH tissue, IL-15, IL-17, IFN-γ, and IL-8 are released by stromal cells, infiltrating T cells, basal and stromal cells, as well as epithelial cells, respectively (76). Following the release of IL-5, IL-6, and IL-8 as chemotactic factors for T cells’ locomotion and infiltration of them to the TME (62, 79). An investigation reported that the levels of TGF-β and IL-6 were decreased by amplified levels of CXCL9, enhancing the growth of tumors in a mouse model of PC. Moreover, overexpression of CXCL9 reduced the T cells frequency in immune organs and the TME and decreased the release of TGF-β2 and IL-6, resulting in the development of PC (80).

On the other hand, IL-17-producing T-cells need IL-23 as an additional factor to survive (81). It has been demonstrated that stromal prostate cells in BPH tissue can act as antigen-presenting cells (APC) and activate antigen-specific CD4^+^ T cells to release IL-17 and IFN-γ, stimulating the release of IL-6 (a potent autocrine growth factor) and IL-8 (a paracrine inducer of fibroblast growth factor 2) which are the main inducer of stromal and epithelial prostate cells growth and proliferation (82). Findings of another study indicate that the serum levels of anti-inflammatory cytokines, including IL-4, IL-6, and IL-10, were elevated significantly, and it was directly associated with increased PSA in hormone-refractory PC patients compared to values in the hormone-sensitive group (83). These remarks suggest that cytokine modulation and their abnormal response could have an essential role in the pathogenesis of hormone-refractory PC.

**Figure 1:**
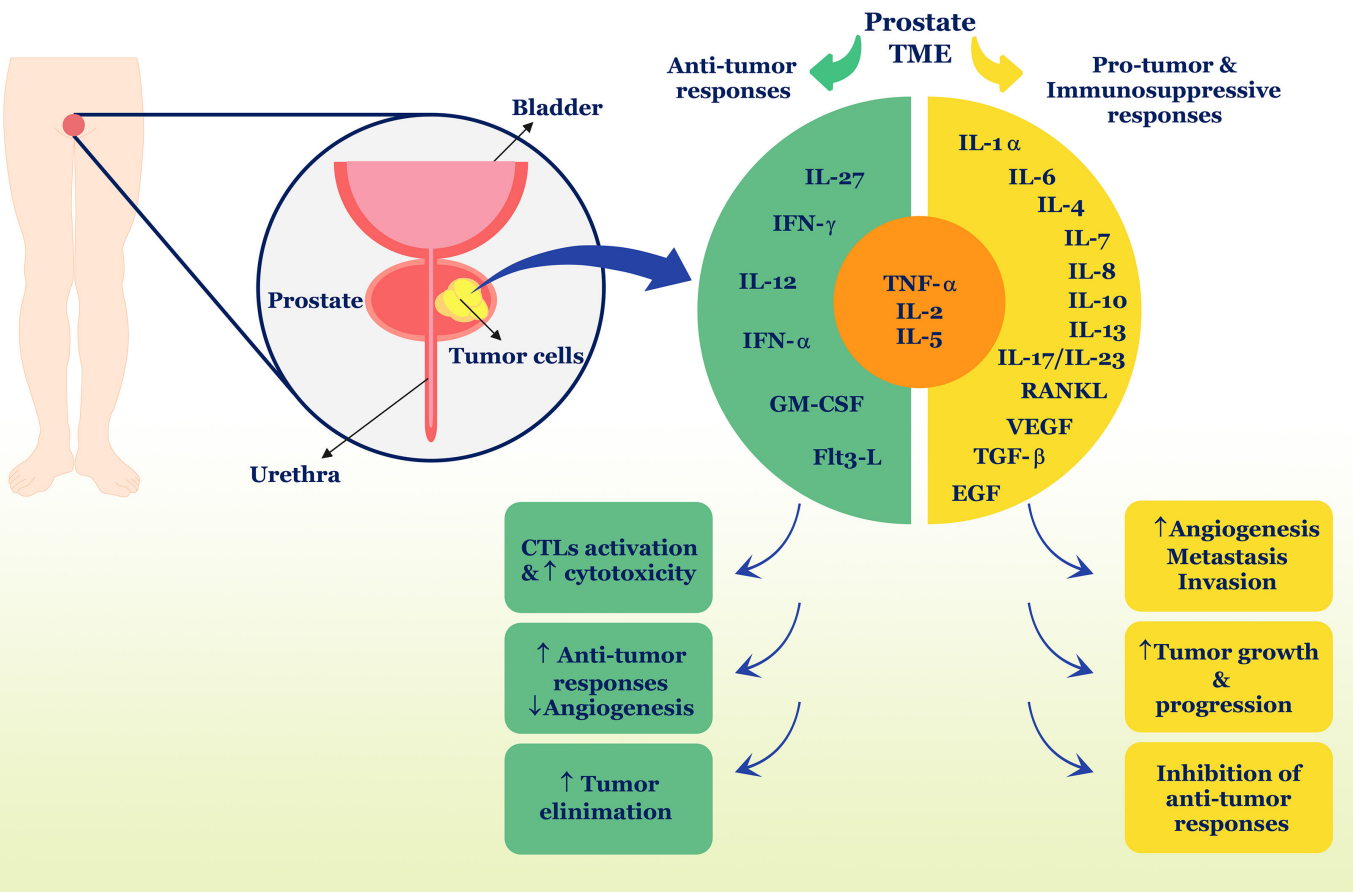
Role of cytokines in PC. Different cytokines can have different roles and properties in the TME and induce anti-tumor responses or suppress anti-tumor responses. In PC, TNF-α, IL-2, and IL-5 can play a dual role and contribute to both types of immune responses. Ultimately, anti-tumor cytokines including IFN-γ, IL-12, IL-27, IFN-α, and GM-CSF, Flt3-L, can stimulate tumor removal processes, but in contrast, pro-tumor cytokines such as IL-1α, IL-6, IL-7, IL-8, IL-10, IL-13, IL-17, IL-23, RANKL, VEGF, TGF-β, and EGF cause tumor development and progression. TME, tumor microenvironment; IL, interleukin; IFN, interferon; GM-CSF, granulocyte-macrophage colony-stimulating factor; TNF, tumor necrosis factor; TGF, tumor growth factor; EGF, epidermal growth factor; CTL, cytotoxic T lymphocyte; flt3-L, Fms-like tyrosine kinase-3 ligand.

Collectively, it can be concluded from this section that cytokines act as a network, increasing the complexity of recognizing their function in different pathological conditions. It seems that in PC, these cytokines can both induce anti-tumor defense by the immune system and cause tumor survival and growth by inhibiting anti-tumor defense or increasing inflammation (**Figure 1**) (**Table 1**). Also, cross-talk between cytokines and sex hormones can play a pivotal role in the pathogenesis of PC.

**Table 1:** Role of cytokines in the pathogenesis and treatment of PC.

Cytokine	Role in PC pathogenesis	Role in PC immunotherapy	Ref
**IL-1α and IL-1β**	Increased in PC Cell proliferation High prostate-specific antigen levels Induces STAMP2 expression IL-1α induces the immunosuppressive function of MSCs		(60, 67, 74)
**IL-2**	Induce of Tregs Tumor growth and progression Anti-tumor properties	Anti-tumor properties Increase cytotoxicity Changes in tumor blood flow NK cell activation Increased following CAR-T cell therapy	(84–89)
**IL-4**	Stimulate prostate epithelial cells to express 3b-HSD Increases the expression of androgens Activation of the JNK pathway Up‐regulation of survivin Tumor progression	Induces CRS following CAR-T cell therapy	(74, 83)
**IL-5**	Induce locomotion and infiltration of T cells into the TME Induce of eosinophils and anti-tumor effects	CRS following CAR-T cell therapy	(62, 79, 90, 91)
**IL-6**	Increased in PC Prognostic biomarker Induces of EMT Cancer metastasis Formation of premetastatic niche CTC attachment to endothelial Stimulates extravasation TME remodeling Alteration of the bone tissue Increase expression of AR Induce locomotion and infiltration of T cells into the TME Induces STAMP2 expression	One of the most critical cytokines in the CRS following CAR-T cell therapy	(63–67, 74, 90, 91)
**IL-7**	Induces of EMT Cancer metastasis		(42)
**IL-8**	Induces of EMT Induces locomotion and infiltration of T cells into the TME Cancer metastasis Angiogenesis	Induces CRS following CAR-T cell therapy	(38, 48, 50, 76, 82, 90, 91)
**IL-10**	Inhibits anti-tumor responses Regulatesg the androgen response	Induces CRS following CAR-T cell therapy	(32, 34, 44, 83, 90, 91)
**IL-12**	Induction of anti-tumor responses p40 is involved in the facilitation process of tumor cell escape p40 is involved in the inhibition of IL-12Rβ1	Stimulating Th1 responses IFN-γ production Anti-tumor effects	(92–96)
**IL-13**	Stimulate prostate epithelial cells to express 3b-HSD Increase expression of androgens		(75)
**IL-17**	Increase expression of PDL-1 Increase expression of COX-2 Induction release of IL-6 and IL-8		(61, 62, 76, 82)
**TNF-α**	Increase expression of PDL-1 Both apoptotic and survival impacts on tumor cells	Anti-tumor effects In patients with LA-HRPC, intra-prostatic administration of a tolerable toxicity dose of TNF-α could help treat these patients	(41, 60, 61, 74, 97–99)
**IL-23**	Regulates the androgen response Th17 survival		(44, 81)
**IL-27**	Anti angiogenic effects Anti-tumor effects		(100)
**flt3-L**	Infiltration of CD4^+^, CD8^+^ T cells, NK-cells and DCs Anti-tumor effects	Induces CRS following CAR-T cell therapy	(90, 91, 101)
**TGF-β**	Induction of EMT Cancer metastasis (bone) Inhibition of anti-tumor responses Reduce the expression of MHC-I Angiogenesis *via* regulating IL-8 expression Formation of premetastatic niche CTC attachment to endothelial Stimulation of extravasation TME remodeling Alteration of the bone tissue		(32, 34, 38, 39, 45, 46, 48, 50–59, 75, 78)
**EGF**	Bone metastasis		(45, 46)
**GM-CSF**	Stimulation of macrophages, DCs, NKT cells, and granulocytes Increase tumor antigens presentation to effector T cells	Induce production of CD4^+^ T cells Development of CD4^+^ and CD8^+^ PAP specific responses Stimulation of anti-PC-antigen responses by effector T and B cell Induces CRS following CAR-T cell therapy	(90, 91, 102–110)
**IFN-α**		Affect tumor cells directly Stimulate immune system components	(111–114)
**IFN-γ**	Induce IL-6 and IL-8 release Induce anti-tumor responses	Induction of anti-tumor responses Increased following CAR-T cell therapy Increased following treatment with DC-vaccine (Provenge) and AST Induces CRS following CAR-T cell therapy	(73, 74, 76, 78, 89–91, 93, 94, 115)
**VEGF**	Angiogenesis Formation of premetastatic niche CTC attachment to endothelial Stimulation of extravasation TME remodeling Mitogenic properties Induce endothelial cell proliferation Tumor invasion and metastasis		(48–53)
**RANKL**	Alteration of the bone tissue Osteoclastogenesis Formation of premetastatic niche CTC attachment to endothelial Stimulation of extravasation TME remodeling		(52, 53, 56–59)

STAMP2, six transmembrane protein of prostate 2; PC, prostate cancer; LA-HRPC, hormone-resistant prostate cancer; Treg, regulatory T cell; CAR-T cell, chimeric antigen receptor T cells; TME, tumor microenvironment; IL, interleukin; IFN, interferon; GM-CSF, granulocyte-macrophage colony-stimulating factor; TNF, tumor necrosis factor; TGF, tumor growth factor; EGF, epidermal growth factor; CTL, cytotoxic T lymphocyte; 3b-HSD, 3β-Hydroxysteroid dehydrogenase/Δ⁵⁻⁴ isomerase; EMT, epithelial–mesenchymal transition; NK cell, natural killer cell; CRS, cytokine release syndrome; AR, androgen receptor; CTC, circulating tumor cells; MHC, major histocompatibility complex; DC, dendritic cell; AST, androgen suppression treatment; COX, cyclooxygenase; PDL-1, programmed death ligand-1; PAP, prostatic-specific acid phosphatase; flt3-L, Fms-like tyrosine kinase-3 ligand; MSCs, mesenchymal stem cells.

## Cytokines and PC Immunotherapies

In recent years, cancer immunotherapy has been one of the most successful therapies in the treatment of cancer, and various immunotherapy-based methods such as cancer vaccines, cell-based therapies, the use of monoclonal antibodies, cytokine therapy and combination therapies have been used in the treatment of PC and some of which have been almost successful (116). In this section, the advantages and disadvantages of cytokines in PC treatment are discussed (**Table 1**).

### Cytokine-Based Immunotherapy

As mentioned before, a blend of different cytokines is presented in the TME, which forms anti-tumor or pro-tumor mechanisms and responses (16). Accordingly, manipulating cytokines with different cancer treatment approaches can cause fluctuations in the TME and have a protective effect against the tumor. However, administrating cytokines requires several considerations. On the other hand, the outcomes of various immunotherapy methods may be affected by the positive or negative effects of cytokines.

In general, cytokine therapy in cancer is performed by systemic administration and direct injection into tumor tissue. It has been reported that interferon-α (IFN-α) has probably been one of the most effective cytokines in the treatment of solid and hematological malignancies to date (111). In this regard, randomized clinical trials recognized that IFN-α decreases the risk of malignant melanoma recurrence subsequent surgical removal of localized lymph node metastasis (112, 113). Although the anti-tumor mechanism of IFN-α is not yet fully understood, it is thought to affect tumor cells directly and stimulate immune system components. A study on PC patients showed that systemic IFN-*a*2b had no systemic anti-tumor effect (97). Additionally, another *in vitro* investigation reported that the treatment of the human prostate cancer LNCaP‐LN3 cell line with IFN-α had no effective anti-tumor outcome (114).

The interleukin-2 infusion can also be used to treat cancer. Studies show that injection of this cytokine in high doses can have anti-tumor properties; although its mechanism of action has not been determined, it probably exerts its anti-tumor properties by increasing cytotoxicity and changes in tumor blood flow (84, 85). Administration of IL-2 in low doses can be less toxic and cause natural killer (NK) cell activation. One of the significant challenges in using IL-2 in cancer treatment is the activation of Tregs because these cells consume IL-2 and are unable to produce it themselves, suppressing anti-tumor responses and causing tumor growth and progression (86). An experimental study used KM2812 as an IL-2 construction and observed that following treatment of mice in some animals, complete regression occurred, and KM2812 had a considerable anti-tumor effect (87). On the other hand, due to Treg activation by IL-2 and its dose adjustment complications in cancer treatment, some studies have shown that T cell depletion is more effective than neutralization of IL-2 in PC (88).

Evidence shows that despite the several side effects of using TNF-α in cancer therapy, beneficial therapeutic effects have been observed in some cancers such as sarcoma and melanoma (98). In patients with hormone-resistant prostate cancer (LA-HRPC), intra-prostatic administration of a tolerable toxicity dose of TNF-α could help treat these patients (97). Treatment of the LNCaP cell line employing iron oxide nanoparticles (IONPs) loaded with soluble TNF-α, and lactonic sophorolipids (LSLs) also had beneficial anti-tumor advantages. It was suggested that combination might have a synergistic effect, exhibiting more effective therapeutic than either compound alone in the treatment of PC (99).

Interleukin-12 is also one of the critical cytokines in anti-tumor defense that can inhibit tumor growth and progression by stimulating Th1 responses and IFN-γ production, increasing the cytotoxic activity of lymphocytes, and inhibiting lymphocytes angiogenesis (92, 117). Evaluation of IL-12 biostructure showed that it is composed of two different subunits termed p40 and p35, and it has been reported that p40 is involved in the facilitation process of tumor cell escape from immune responses and also cell death as well as the arrest of IL-12Rβ1 but not IL-12Rβ2 (93). To prove these claims, an anti-p40 monoclonal antibody was used, and the findings showed that tumor cell death was increased and the IL-12Rβ1 was activated, and IL-12/IFN-γ down-stream signaling pathway was initiated, resulting in cancer cell death (93). Studies in animal models of cancer show that systemic administration of IL-12 as well as viral vectors expressing IL-12 can have significant anti-tumor effects. However, the unknown and hidden dimensions of toxicity and other side effects have led to preliminary human studies (92, 94). An experimental investigation hypothesized that combining the direct oncolytic and anti-angiogenic activities of the IL-12 expressing NV1042 oncolytic herpes simplex virus with microtubule disrupting agents could be an effective method to improve anti-tumor efficiency in the treatment of PC (95, 96).

Granulocyte-macrophage colony-stimulating factor (GM-CSF) is a monomeric glycoprotein produced by mast cells, T cells, macrophages, NK cells, fibroblasts, and endothelial cells that act as a cytokine in the immune system (118). GM-CSF can stimulate macrophages, DCs, NKT cells, and granulocytes activation, improving tumor antigens presentation to effector T cells (102, 103). Additionally, GM-CSF-based vaccines can also produce CD4+ T cells that produce a wide range of cytokines (104). Previous studies on cancer immunotherapy using cytokines showed that the systemic administration of GM-CSF had some clinical benefits in treating PC, pulmonary metastases, and melanoma, perhaps *via* stimulation of the immune system anti-tumor responses (105).

Although type Th2-type cytokines such as IL-4, IL-5, IL-6, IL-10, and IL-13 are more involved in host anti-parasitic defense and allergic reactions, evidence suggests that they can elicit anti-tumor responses *via* eosinophils activation as well as stimulating antibody production. However, activating Th2-dependent responses may not be as desirable as Th1 responses in cancer immunotherapy (104, 119). In this regard, a study on a PC mouse-xenografts model showed that IL‐4 could stimulate the activation of the JNK pathway and the up‐regulation of survivin (an anti-apoptotic protein), progressing PC (120).

Evidence has revealed that IL-27, an immune regulator cytokine, can contribute to anti-tumor responses in several cancers without noticeable toxicity. In a mouse model of PC treated by IL-27, it has been revealed that this cytokine therapy can inhibit tumor cell growth and proliferation, reducing tumor size. The anti-tumor activity of IL-27 inhibits angiogenesis and shifts the phenotype of tumor cells to anti-angiogenic, which disrupts the formation of microvascular networks and inhibits tumor growth and invasion (100). Interestingly, in normal epithelial tissue of patients with PC, IL-27 receptor (IL-27R) expression is high, but in low-grades of PC, the expression of IL-27R is relatively lower than normal tissue, and in high-grade tumor tissue, its expression is completely stopped. However, the expression of IL-27R on infiltrated CD11c^+^ immune cells and CD4+ and CD8+ lymphocytes in the TME is still high, which upon binding of IL-27 to the receptors can trigger antitumor responses (100).

Previous studies showed that Fms-like tyrosine kinase-3 ligand (flt3-L), a hematopoietic four helical bundle cytokine, can induce remarkable growth inhibition of prostate tumor cells that grow at an ectopic site. In flt3-L-treated severe combined immunodeficient (SCID) mice, the growth of tumor stabilization and regression of palpable ectopic prostate tumors (TRAMP-C1) cells was more rapid than in flt3-L-treated wild-type mice. These findings indicated that innate and adaptive immune responses are involved in tumor growth inhibition mediated by flt3-L. It has been revealed that flt3-L therapy can lead to infiltration of CD4^+^, CD8^+^ T cells, NK-cells and dendritic cells (DCs) in the TME. However, it has been shown that tumor-associated DCs, as well as the immunosuppressive milieu of the TME, can attenuate the effects of Flt3-L and cause treatment to fail (101). It has been revealed that the six transmembrane protein of prostate 2 (STAMP2), a significant factor for PC cells growth and survival and its expression is regulated through inflammatory signaling. In this regard, IL-1β and IL-6 can synergistically induce STAMP2 expression. Fascinatingly, the knockdown of STAMP2 can increase PC cells’ sensitivity to cytokine therapy. Therefore, inflammatory-mediated regulation of the STAMP2 can affect PC progression (74).

Collectively, administration of the cytokines due to cancer treatment can have significant side effects, just like when the body has a severe infection. Furthermore, systemic cytokine administration has had few therapeutic consequences due to the pleiotropic effects of cytokines in the TME.

### Role of Cytokines in Other PC Immunotherapy Approaches

Phase I clinical trial study on biochemically recurrent PC using DNA vaccines that encode prostatic acid phosphatase (PAP) plus GM-CSF (pTVG-HP) showed that the vaccination was safe, and CD4^+^ and/or CD8^+^ PAP specific responses were developed in some patients (106). In this context, another phase I trial study reported that a retroviral-based vaccine expressing GM-CSF could stimulate anti-PC-antigen responses by effector T and B cells (107). Although GM-CSF cellular vaccines are not used in the clinic for PC, this cytokine is widely used in combination therapies and other PC vaccines in preclinic studies and research on GM-CSF in PC treatment is ongoing (108). For instance, a phase II clinical trial study that used PROSTVAC [an active immunotherapy vaccine that contains prostate-specific antigen (PSA)] and GM-CSF in a group of men with metastatic castration-resistant PC showed an increased survival of patients (109). Similar studies have also shown that PROSTVAC alone may not be effective because immunosuppressive mediators and cytokines inhibit vaccine-induced anti-tumor responses in the TME; nonetheless, when co-administered with GM-CSF, it can induce a more effective anti-tumor response (110).

Another therapeutic approach in PC leading to stimulate cytokines is reovirus therapy. It has been shown that following reovirus therapy, the secretion of pro-inflammatory cytokines as well as CD8^+^ T and NK cell trafficking and infiltration is increased in the TME, and it can lead to increased expression of MHC-I on the surface of tumor cells to recognize by APCs and further tumor antigen presentation to effector lymphocytes (121, 122).

As mentioned before, androgens play an essential role in the development of PC. Accordingly, androgen suppression therapy (AST) has always been an effective treatment in metastatic castration-resistant PC by modulating tumor cells’ sensitivity to T cells and accumulation of effector CTLs into the TME in PC (123). Evidence demonstrated that the tumor progression properties of hormone-refractory PC might be changed by direct cytokine therapy (124). The status of the immune system can determine the response to hormone therapy, and it can also change with age. Accordingly, assessing the balance between pro-inflammatory (Th1) and anti-inflammatory (Th2) cytokines in response to hormone therapy is essential. To increase the effectiveness of cancer therapy, combination therapies including immunotherapy and AST have recently been used, and a clinical trial in this field showed that administration of DC-vaccine (Provenge) before AST led to an increase of effective cytokines such as IFN-γ along with tumor-specific T cells in men with hormone-sensitive patients biochemically recurrent prostate cancer PC (115). Therefore, it can be concluded that cytokines play a positive and anti-tumor role in this therapeutic approach.

The development of chimeric antigen receptor (CAR)-T cell therapy has taken essential steps in treating cancer, especially blood malignancies (90, 125). Recently, this immunotherapy method has been used in the treatment of prostate cancer. A study used CAR-T cell-specific for PSMA that was resistant to TGF-β by infecting CD8^+^ T cells obtained from metastatic castration-resistant PC (mCRPC) patients with a retroviral construct in a mouse xenograft model, and the findings showed that CAR-T cells could lysis the tumor PSMA^+^ PC3 cells but not PSMA^-^ PC3 tumor cells. Interestingly, apoptosis of the tumor cells, infiltration of CD8^+^ cells, as well as amplified IFN-γ and IL-2 levels were only realized in PSMA^+^ PC3 tumor cells (89). These findings suggest that CAR-T cells can have positive therapeutic effects in treating PC, eliminating tumor cells expressing specific antigens and increasing levels of cytokines that are effective in anti-tumor defense. Although CAR-T cell therapy can successfully treat cancer, hematological trials reported that CAR-T cell therapy could also have various adverse effects and toxicities, one of the most important of which is cytokine release syndrome (CRS) (126–129). Fever and even lethal capillary leakage, along with hypotension and hypoxia, are the primary manifestation of the CRS. CRS is the specific spectrum of reactions realized following administration of targeted therapies such as CAR T cell therapy that cause remarkable activation of the immune system. The onset of clinical signs of CRS is delayed and indicates that these clinical signs, such as fever, seek T cells activation and proliferation due to the identification of targeted tumor antigens. It has been revealed that the CRS is detected in patients undergoing CAR-T cell therapy and patients receiving blinatumomab (130).

Moreover, higher serum levels of INF-γ, FLT3-ligand, CX3CL1, IL-5, IL-6, IL-8, IL-10, and GM-CSF were detected in patients with severe CRS upon CAR-T cell therapy (90, 91). Evidence demonstrated that using an anti-IL-6 receptor antagonist (Tocilizumab) is considered standard therapy for CRS management; however, the administration’s timing is crucial and has not yet been determined precisely (131). The outcomes of investigations in PC treatment using tumor-specific antigen CAR-T cells were shown to have limited side effects and systemic immune-related adverse events (IRAEs). There are various tactics to reduce the IRAEs and toxicity of CAR-T cell therapy, including combining high-affinity anti-PSMA and low-affinity anti-PSCA CAR-T cells, inserting a suicide gene to remove hyperactivated CAR-T cells, using other cellular sources such as NK cell, and also using nanoparticles for the delivery of CAR-T cells are proposed (132–137).

Recently, an investigation demonstrated that the long non-coding RNA (lncRNA) OGFRP1 could be involved in inducing malignancy in PC. In this context, OGFRP1 is able to sequester miR-149-5p, stimulating IL-6 upregulation and promoting chemoresistance in PC tumor cells. Therefore, the OGFRP1/miR-149-5p/IL-6 axis may be a potential therapeutic target for treating chemoresistance PC cells (138).

## Concluding Remarks

According to the findings of the discussed studies, cytokines, as an important group of immune system mediators, appear to play a significant and dual role in the pathogenesis of PC. Contrary to our knowledge about the role of different cytokines in the pathogenesis of cancers, some inflammatory and immunosuppressive cytokines can lead to PC progression. On the other hand, sex hormones and cross-talk between these hormones and cytokines can be involved in the pathogenesis of PC. Moreover, in PC immunotherapy, cytokines can also affect the outcome of treatment. Treatment with cytokines and their administration has also had various effects due to the pleiotropic effect of cytokines on the TME and, depending on different pathological conditions, complicating their role. It seems that more studies should be done on these multifunctional molecules in order to adopt a more appropriate strategy for the treatment of PC.

## Author Contributions

CM: Conception, design and inviting co-authors to participate. YD: Writing original manuscript draft. NX: Review and editing of manuscript critically for important intellectual content and provided comments and feedback for the scientific contents of the manuscript. All authors contributed to the article and approved the submitted version.

## Funding

This work was supported by the Zhejiang Provincial Natural Science Foundation of China (No. LQ19H160027 to CM).

## Conflict of Interest

The authors declare that the research was conducted in the absence of any commercial or financial relationships that could be construed as a potential conflict of interest.

## Publisher’s Note

All claims expressed in this article are solely those of the authors and do not necessarily represent those of their affiliated organizations, or those of the publisher, the editors and the reviewers. Any product that may be evaluated in this article, or claim that may be made by its manufacturer, is not guaranteed or endorsed by the publisher.
